# Tissue transglutaminase-2 promotes gastric cancer progression *via* the ERK1/2 pathway

**DOI:** 10.18632/oncotarget.6883

**Published:** 2016-01-11

**Authors:** Xiaofeng Wang, Zhenjia Yu, Quan Zhou, Xiongyan Wu, Xuehua Chen, Jianfang Li, Zhenggang Zhu, Bingya Liu, Liping Su

**Affiliations:** ^1^ Shanghai Key Laboratory of Gastric Neoplasms, Shanghai Institute of Digestive Surgery, Department of Surgery, Ruijin Hospital, Shanghai Jiao Tong University School of Medicine, Shanghai, People's Republic of China

**Keywords:** TG2, gastric cancer, ERK1/2, tumor progression

## Abstract

Gastric cancer (GC) is one of the most common tumors worldwide and involves extensive local tumor invasion, metastasis, and poor prognosis. Understanding mechanisms regulating progression of GC is necessary for developing effective therapeutic strategies. Tissue transglutaminase-2 (TG2), a multifunctional member of the transglutaminase family, has been shown to be critical for tumor initiation and progression. However, how TG2 promotes the progression of GC is unknown. We report that TG2 was highly expressed in GC tissues and positively associated with depth of tumor invasion and late TNM stage. With gain- and loss-of-function approaches, we observed that TG2 promoted GC cell proliferation, migration, invasion, as well as tumorigenesis and peritoneal metastasis *in vivo*. These events were associated with the ERK1/2 pathway activation and an ERK1/2 inhibitor (U0126) inhibited cell proliferation, migration, and invasion induced by overexpression of TG2. In summary, TG2 contributes to tumorigenesis and progression of GC by activating the ERK1/2 signaling pathway and is a potential therapeutic target of metastatic gastric cancer.

## INTRODUCTION

Gastric cancer (GC) is one of the most frequent tumors worldwide and the third leading cause of tumor death according to GLOBOCAN 2012 [1]. Although appropriate surgical resection and adjuvant treatments such as radiotherapy and chemotherapy are used to treat GC, 5-year survival is less than 20% [2] due to local tumor invasion, systemic dissemination, metastases, and recurrence. Therefore, a better understanding of molecular mechanisms accounting for GC development and progression could contribute to prevention, diagnosis, and treatment of GC.

Tissue transglutaminase-2 (TG2 or tTG), the most complex member of the transglutaminase (TGase) family, is a calcium-dependent cross-linking enzyme that catalyzes protein modifications *via* transamidation to facilitate formation of lysine combinations or polyaminated proteins in the presence of calcium [3, 4]. TG2 is expressed ubiquitously and abundantly in various subcellular areas including the cytosol, nucleus, cellular membrane, and the extracellular matrix (ECM) [5-7]. TG2 is a multifunctional molecule that can bind to GTP and has hydrolysis [8], protein disulfide isomerase [9], and protein kinase activity independent of calcium [10]. Furthermore, TG2 has calcium-independent non-enzymatic activity, interacting with many cell surface proteins [11], participating in inflammation, differentiation, apoptosis, cell migration, wound healing, neurodegenerative disorder, and cancer [12-15].

Recently, accumulated evidence indicates that TG2 is involved in tumor formation and progression by organizing the ECM, regulating cancer cell adhesion to the endothelium, as well as controlling migration and invasion of cancer cells and angiogenesis of tumor tissue [16]. Elevated TG2 expression has been observed in breast [17], pancreatic [18], colon [19], lung [20] and ovarian cancers [21], and it has been correlated with cell survival and high tumor invasiveness. For example, in non-small cell lung cancer, progression-free survival (PFS) of high-expressing TG2 patients was shorter than that of low-expressing TG2 patients [20]. In addition, TG2 promoted ovarian tumor metastasis by inducing a cancer stem cell phenotype and epithelial-to-mesenchymal transition (EMT) [22]. Even so, a precise role for TG2 in the development and progression of GC has not been well defined.

Here we measured TG2 expression in GC tissues and corresponding non-tumor mucosal tissues and explored the role and underlying mechanism of TG2 with respect to GC progression using *in vitro* and *in vivo* models. TG2 expression was frequently elevated in GC and associated with tumor depth of invasion and late TNM stage. In addition, TG2 promoted GC cell proliferation, migration, and invasion *in vitro*, as well as tumorigenesis and peritoneal metastasis *in vivo* through activation of the ERK1/2 pathway in GC cells. Thus, TG2 is a potential therapeutic target for treatment of metastatic GC.

## RESULTS

### TG2 expression is upregulated in GC cells and associated with clinicopathology

**Figure 1:**
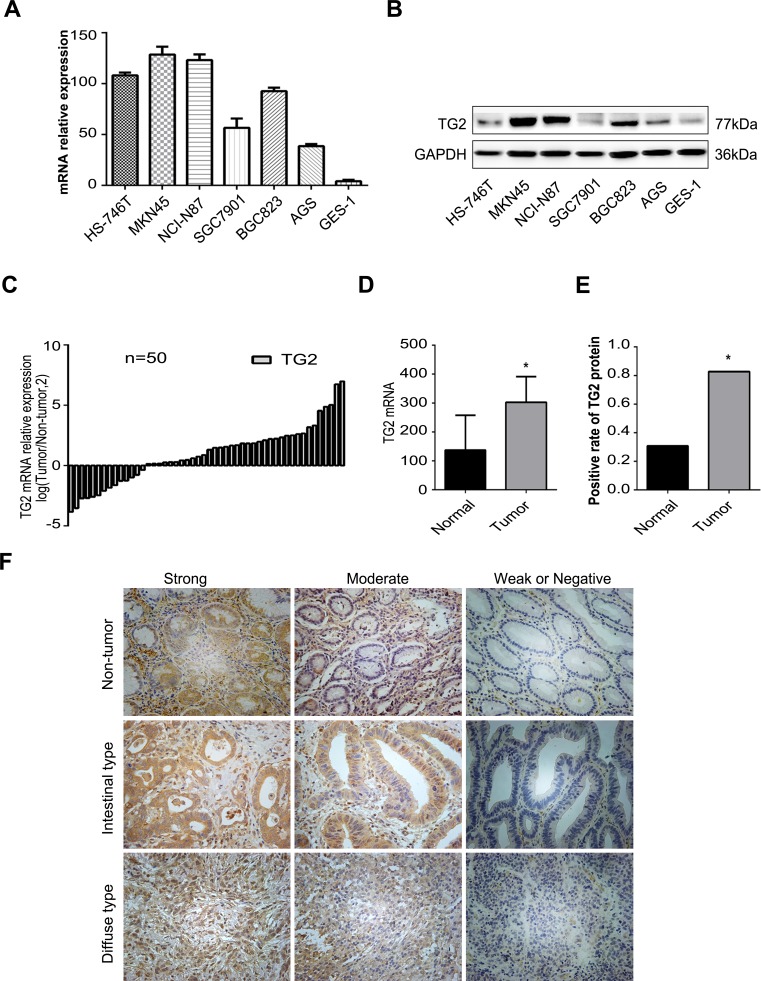
Expression of TG2 in the GC tissues and cell lines **A.** TG2 mRNA expression in GC cell lines and a normal gastric epithelial cell line GES-1 was analyzed by qRT-PCR. **B.** TG2 protein expression in human GC cell lines and a normal gastric epithelial cell line GES-1 was examined by Western blot. **C**. and **D**. TG2 mRNA expression in 50 gastric cancer tissues and adjacent non-tumor tissues was analyzed by qRT-PCR. Data are 2^−ΔCt^ (*P < 0.05). **E.** Positive ratios of TG2 protein expression in 127 pairs of gastric cancer tissues. **F.** Expression of TG2 was examined with immunohistochemistry (IHC) in non-tumor gastric tissues, intestinal-type GC tissues, and diffuse-type GC tissues, and classified as strong expression (++), moderate expression (+), weak positive or negative expression (± or −). Original magnification: 200x.

To elucidate the role of TG2 in human GC, we measured TG2 expression in human GC cell lines and GC tissues. TG2 mRNA in six GC cell lines, one normal gastric epithelial cell line (GES-1), and 50 pairs of human GC and matched adjacent non-tumor tissues was measured with qRT-PCR. As shown in Figure 1A, TG2 mRNA expression was significantly up-regulated in GC cell lines compared with normal GES-1 cells. Moreover, TG2 mRNA in GC tissues was higher than in non-tumor tissues (Figure 1C and 1D) and this finding was confirmed with Western blot (Figure 1B).

Upregulated TG2 expression was assayed in 127 GC and matched non-tumor tissue pairs using immunohistochemistry (IHC) as depicted in Methods. Of all GC tissues, 82.7% were positive for TG2 protein expression and 22 GC tissues and 88 adjacent normal tissues were negative for TG2 protein or had weak expression (Figure 1E; *P* < 0.05). Typical immunostaining of TG2 in normal and GC tissues was shown in Figure 1F and positive TG2 protein staining occurred in the cytoplasm of GC cells. Elevated TG2 protein expression in tumor tissues was significantly associated with depth of tumor invasion (*P* = 0.026) and late TNM stage (*P* = 0.011), but not with other parameters (see Table 1). Thus, TG2 expression is up-regulated in GC cells and is associated with tumor severity.

**Table 1:** Association between TG2 expression and clinicopathological parameters in 127 pairs of GC tissues

Variables	Number of cases	TG2 immunostaining			*P* value
		++(n=67)	+(n=38)	±or −(n=22)	
Gender					
male	86	49	24	13	0.365
female	41	18	14	9	
Age(years)					
<60	59	32	21	6	0.106
>60	68	35	17	16	
Tumor size(cm)					
<5	37	19	12	6	0.92
>5	90	48	26	16	
Differentiation					
Poorly, undifferentiated	98	54	30	14	0.246
Well, moderately	29	13	8	8	
T stage					
T1+T2	29	9	12	8	0.026*
T3+T4	98	58	26	14	
Lymphnode metastasis					
Negative	37	14	13	10	0.063
Positive	90	53	25	12	
Distant metastasis					
Negative	97	55	27	15	0.269
Positive	30	12	11	7	
TNM stage					
I+II	46	17	16	13	0.011*
III+IV	81	50	22	9	
Lauren classification					
Intestinal	55	31	16	8	0.707
Diffuse	72	36	22	14	

### TG2 promotes GC cell proliferation

**Figure 2:**
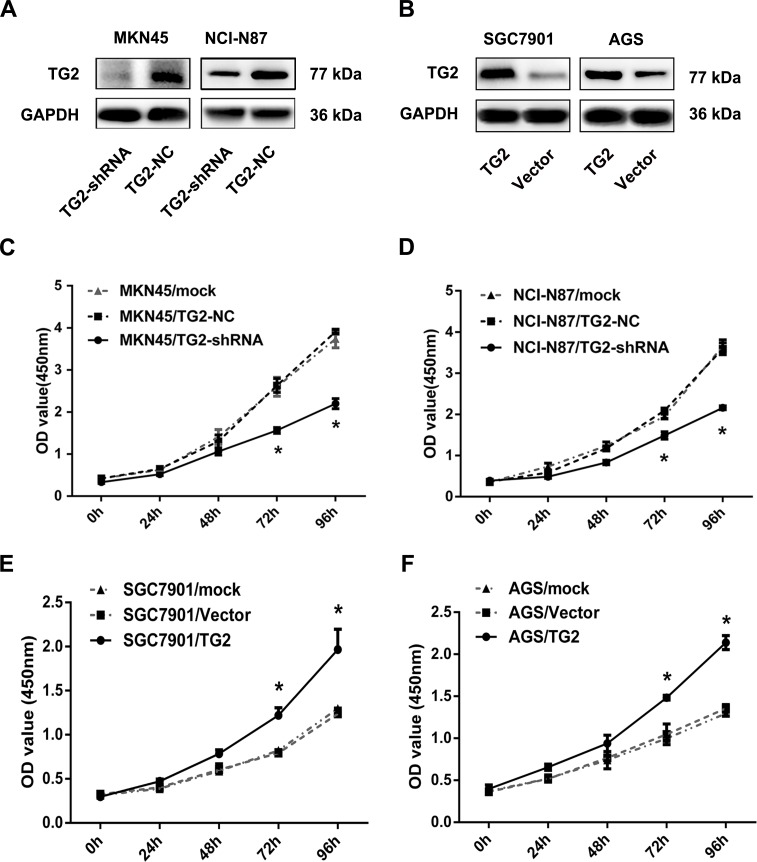
TG2 promotes GC cell proliferation **A.** TG2 protein expression in MKN45 and NCI-N87 cells transfected with TG2-shRNA was quantified with Western blot. **B.** TG2 protein expression in SGC7901 and AGS cells transfected with TG2-constructed plasmid. **C.** and **D.** The effect of TG2 knockdown on MKN45 and NCI-N87 cell proliferation was measured by CCK8 assay. **E.** and **F.** The effect of TG2 overexpression on SGC7901 and AGS cell proliferation was measured by CCK8 assay. Data are means ± SD of three independent experiments (*P < 0.05).

Given that TG2 is frequently overexpressed in GC, it may act as an oncogene. To study this, we measured cell growth in cells that variously expressed TG2. TG2 was highly expressed in GC cell lines compared with a normal gastric epithelial cell line GES-1. We also silenced TG2 in MKN45 and NCI-N87 cells using small interfering RNA. TG2-shRNA was transfected into MKN45 and NCI-N87 cells to knock down TG2 expression and Figure 2A showed that TG2 decreased in TG2-shRNA-transfected cells. With TG2 expression modification confirmed, we measured cell proliferation and noted that proliferation in both MKN45/TG2-shRNA and NCI-N87/TG2-shRNA cells was slower than in negative controls and mock groups (Figure 2C and 2D). Additionally, a TG2-expressing plasmid vector was transfected into SGC7901 and AGS cells, which expressed less TG2 (Figure 2B). We noted that SGC7901/TG2 and AGS/TG2 cells grew faster than vector and mock groups (Figure 2E and 2F). Thus, TG2 promotes GC cell proliferation.

### TG2 enhances migration and invasion of GC cells

**Figure 3:**
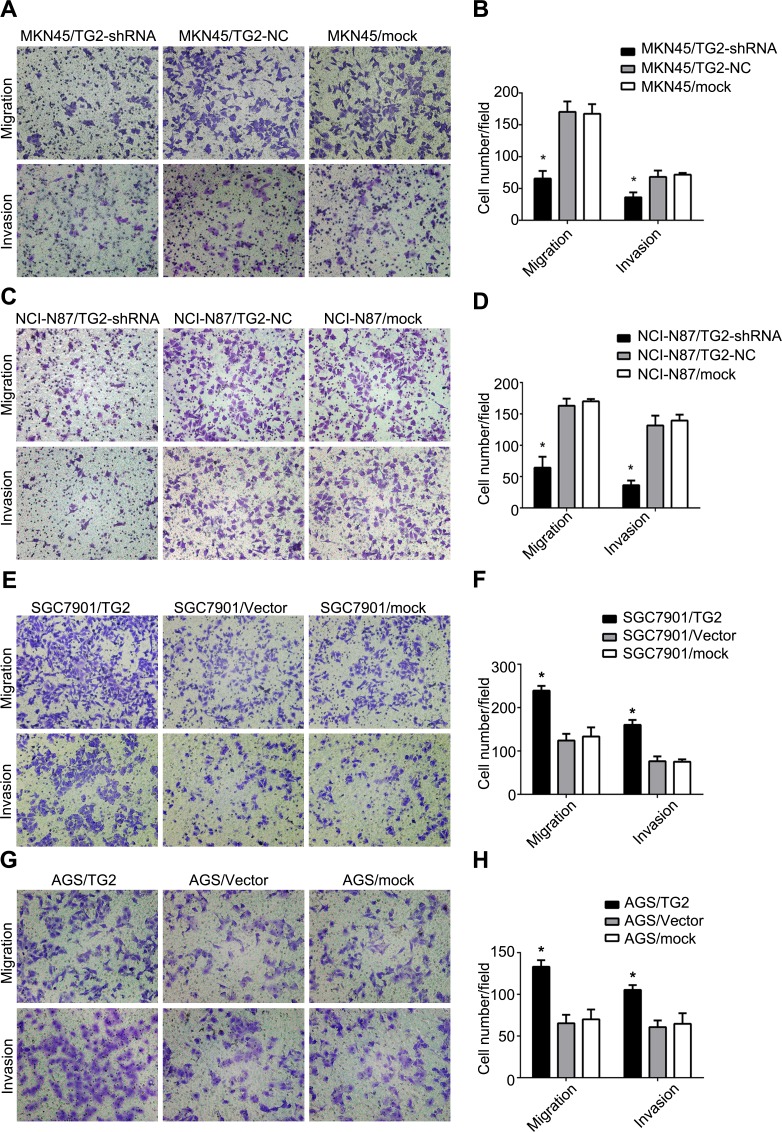
TG2 enhances migration and invasion of GC cells **A.**, **C.**, **E.**, and **G.** Effect of knockdown and overexpression of TG2 on cell migration and invasion was assayed and representative photographs of migrating or invaded cells on membranes with or without Matrigel (magnification, 100×) are shown. **B.**, **D.**, **F.**, and **H.** Histograms depict migrating and invaded cells. Cells were counted in five randomly selected microscopic fields. Data are means ± SD of three independent experiments, *P < 0.05.

We assessed TG2 on GC cell migration and invasion, which are key determinants of malignant progression and metastasis, using Transwell assays. As shown in Figure 3A and 3B, knockdown of TG2 decreased migration (MKN45/TG2-shRNA: 65.60±5.37 cells per field; MKN45/TG2-NC: 170.20±7.40 cells per field; MKN45/mock: 167.20±6.78 cells per field; MKN45/TG2-shRNA *vs*. MKN45/TG2-NC or MKN45/mock: *P* < 0.05) and invasion (MKN45/TG2-shRNA: 36.20±3.48 cells per field; MKN45/TG2-NC: 68.20±4.52 cells per field; MKN45/mock: 71.80±1.16 cells per field; MKN45/TG2-shRNA *vs*. MKN45/TG2-NC or MKN45/mock: *P* < 0.05) of MKN45 cells. Similarly, in NCI-N87 cells, knockdown of TG2 decreased migration (NCI-N87/TG2-shRNA: 52.00±2.67 cells per field; NCI-N87/TG2-NC: 163.00±5.04 cells per field; NCI-N87/mock: 170.00±1.64 cells per field; NCI-N87/TG2-shRNA *vs*. NCI-N87/TG2-NC or NCI-N87/mock: *P* < 0.05) and invasion (NCI-N87/TG2-shRNA: 42.20±5.09 cells per field; NCI-N87/TG2-NC: 131.60±7.00 cells per field; NCI-N87/mock: 139.40±4.19 cells per field; NCI-N87/TG2-shRNA *vs*. NCI-N87/TG2-NC or NCI-N87/mock: *P* < 0.05) (Figure 3C and 3D). Consistent with these observations, the number of cells migrating through the Transwell chamber in the TG2-overexpressed group was significantly higher than in the vector and mock groups for both SGC7901 and AGS cells (SGC7901/TG2: 239.00±11.00 cells per field; SGC7901/Vector: 124.00±15.52 cells per field; SGC7901/mock: 133.33±21.03 cells per field; SGC7901/TG2 *vs*. SGC7901/Vector or SGC7901/mock: *P* < 0.05; AGS/TG2: 133.00±7.94 cells per field; AGS/Vector: 65.33±10.12 cells per field; AGS/mock: 70.00±11.79 cells per field; AGS/TG2 *vs*. AGS/Vector or AGS/mock: *P* < 0.05). Similar data were obtained in invasion assays with SGC7901 and AGS cells (SGC7901/TG2: 160.00±11.53 cells per field; SGC7901/Vector: 76.33±11.37 cells per field; SGC7901/mock: 75.00±5.57 cells per field; SGC7901/TG2 *vs*. SGC7901/Vector or SGC7901/mock: *P* < 0.05; AGS/TG2: 105.33±5.69 cells per field; AGS/Vector: 60.07±8.02 cells per field; AGS/mock: 64.67±12.66 cells per field; AGS/TG2 *vs*. AGS/Vector or AGS/mock: *P* < 0.05) (Figure 3E, 3F, 3G, and 3H). Thus, TG2 enhances migration and invasion of GC cells.

### TG2 regulates cell proliferation, migration, and invasion *via* activation of the ERK1/2 pathway

**Figure 4:**
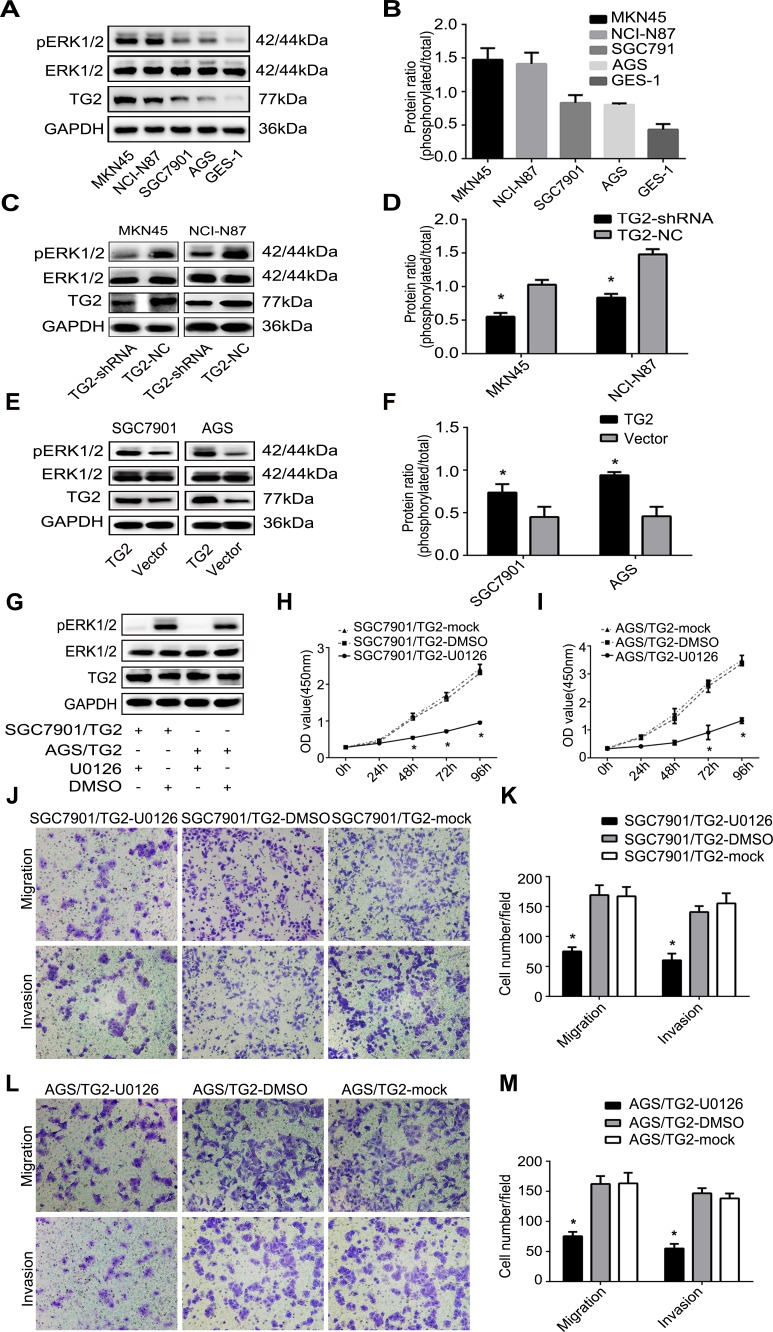
TG2 regulates cell proliferation, migration, and invasion *via* activation of the ERK1/2 pathway **A.** and **B.** ERK1/2 phosphorylation and TG2 expression in GC cells (MKN45, NCI-N87, SGC7901, AGS, and GES-1) were quantified by Western blot. **C.** and **E.** Effects of TG2 on ERK1/2 and phosphorylation were measured. **D.** and **F.** Protein ratios of pERK1/2 in MKN45, NCI-N87, SGC-7901, and AGS cells. **G.** After treatment with U0126 at indicated times, ERK1/2 phosphorylation and TG2 expression in SGC7901/TG2 cells and AGS/TG2 cells were quantified. **H.** and **I.** SGC7901/TG2 and AGS/TG2 cell proliferation were assayed in the presence of U0126. **J., K.**, **L.** and **M.** Effect of U0126 on cell migration and invasion was assayed and typical photographs (magnification, 100×) and migrating or invaded cells are shown. Data are means ± SD of three independent experiments, *P < 0.05.

The ERK1/2 signaling pathway has been reported to be involved in cell growth [16] and migration [23]. Thus, we measured TG2 and assessed whether it could modulate activation of the ERK1/2 signaling pathway in GC cells. Figure 4A and 4B showed that ERK1/2 phosphorylation in MKN45 and NCI-N87 cells, which overexpressed TG2, was greater than in SGC7901 and AGS cells, which expressed less TG2. In contrast, TG2 knockdown significantly inhibited phosphorylation of ERK1/2 in MKN45/TG2-shRNA and NCI-N87/TG2-shRNA cells (Figure 4C and 4D) and TG2 overexpression promoted phosphorylation of ERK1/2 in SGC7901/TG2 and AGS/TG2 cells (Figure 4E and 4F). Therefore, TG2 may be involved in the ERK1/2 pathway regulation as an upstream molecule. We next investigated whether TG2 could regulate cell proliferation, migration, and invasion *via* the ERK1/2 pathway by treating SGC7901/TG2 and AGS/TG2 cells with an ERK1/2 inhibitor (U0126) (20 μM). Western blot and cell proliferation assays indicated that (Figure 4G) ERK1/2 was inhibited, TG2 expression did not change, and SGC7901/TG2 and AGS/TG2 cells growth decreased after treatment with U0126 compared with control or mock cells (Figure 4H and 4I). In addition, after treatment with U0126, migration (SGC7901/TG2-U0126: 75.00±7.00 cells per field; SGC7901/TG2-DMSO: 169.00±16.64 cells per field; SGC7901/TG2-mock: 167.00±15.62 cells per field; SGC7901/TG2-U0126 *vs*. SGC7901/TG2-DMSO or SGC7901/TG2-mock: *P <* 0.05) and invasiveness (SGC7901/TG2-U0126: 60.33±11.06 cells per field; SGC7901/TG2-DMSO: 140.67±10.07 cells per field; SGC7901/TG2-mock: 155.33±16.80 cells per field; SGC7901/TG2-U0126 *vs*. SGC7901/TG2-DMSO or SGC7901/TG2-mock: *P <* 0.05) of SGC7901/TG2 cells were significantly decreased (Figure 4J and 4K) and AGS/TG2 cells migrated (AGS/TG2-U0126: 75.33±7.23 cells per field; AGS/TG2-DMSO: 162.33±13.05 cells per field; AGS/TG2-mock: 163.33±17.56 cells per field; AGS/TG2-U0126 *vs*. AGS/TG2-DMSO or AGS/TG2-mock: *P* < 0.05) less and were less invasive (AGS/TG2-U0126: 55.00±7.81 cells per field; AGS/TG2-DMSO: 146.67±8.62 cells per field; AGS/TG2-mock: 138.33±8.02 cells per field; AGS/TG2-U0126 *vs*. AGS/TG2-DMSO or AGS/TG2-mock: *P* < 0.05) (Figure 4L and 4M). Therefore, TG2 regulates cell proliferation, migration, and invasion *via* activation of the ERK1/2 pathway, which contributes to the development and progression of human GC.

### TG2 facilitates tumor growth, peritoneal spread, and metastasis *in vivo*

**Figure 5:**
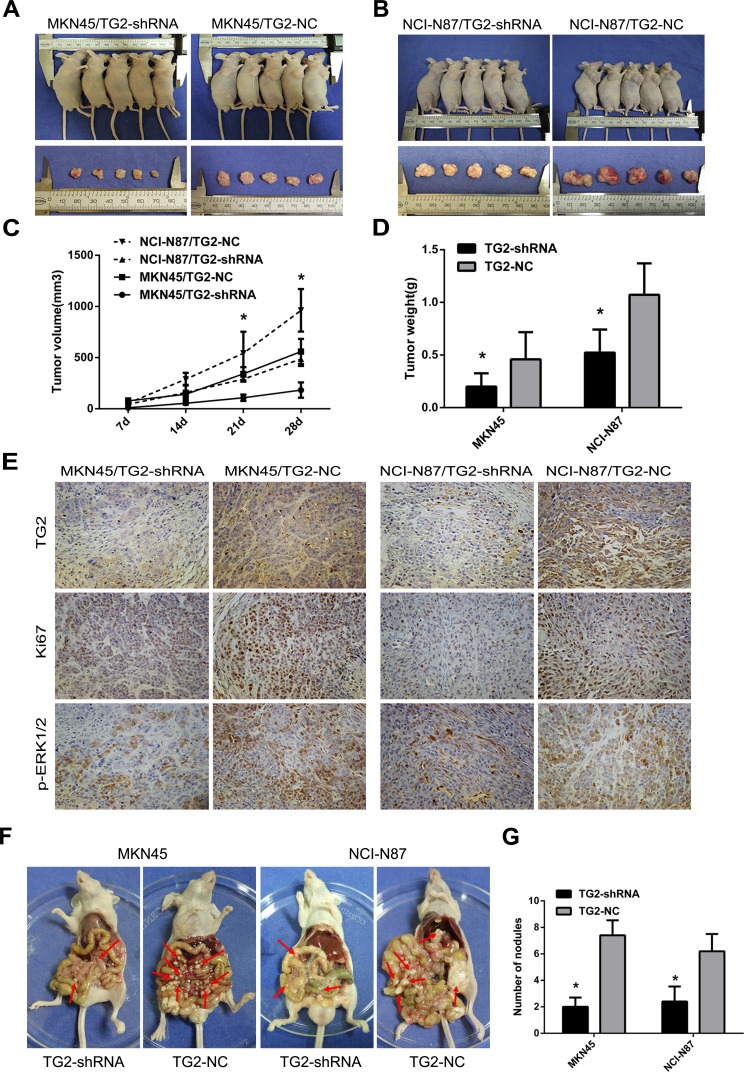
Knockdown of TG2 expression inhibits subcutaneous tumor growth and peritoneal and other metastases in nude mice **A.** and **B.** Representative photographs of tumors in nude mice (*N* = 5 per group) derived from MKN45/TG2-shRNA, MKN45/TG2-NC, NCI-N87/TG2-shRNA, and NCI-N87/TG2-NC cells. **C.** Tumor volume was monitored weekly after treatment with MKN45/TG2-shRNA, MKN45/TG2-NC, NCI-N87/TG2-shRNA, and NCI-N87/TG2-NC cells in nude mice (*P < 0.05). **D.** Average tumor weights derived from MKN45/TG2-shRNA, MKN45/TG2-NC, NCI-N87/TG2-shRNA, or NCI-N87/TG2-NC cells (N = 5) (*P < 0.05). **E.** Expression of TG2, Ki67, and pERK1/2 was quantified by IHC in tumor grafts from MKN45/TG2-shRNA, MKN45/TG2-NC, NCI-N87/TG2-shRNA, or NCI-N87/TG2-NC cells. Original magnification: 200x. **F.** MKN45/TG2-shRNA, MKN45/TG2-NC, NCI-N87/TG2-shRNA, or NCI-N87/TG2-NC cells were inoculated into nude mice, and the peritoneal nodules (red arrows) were observed after 35 days (*N* = 5 per group). **G.** Average peritoneal nodules from nude mice are shown (*P < 0.05). Data are means ± SD of three independent experiments, *P < 0.05.

**Figure 6:**
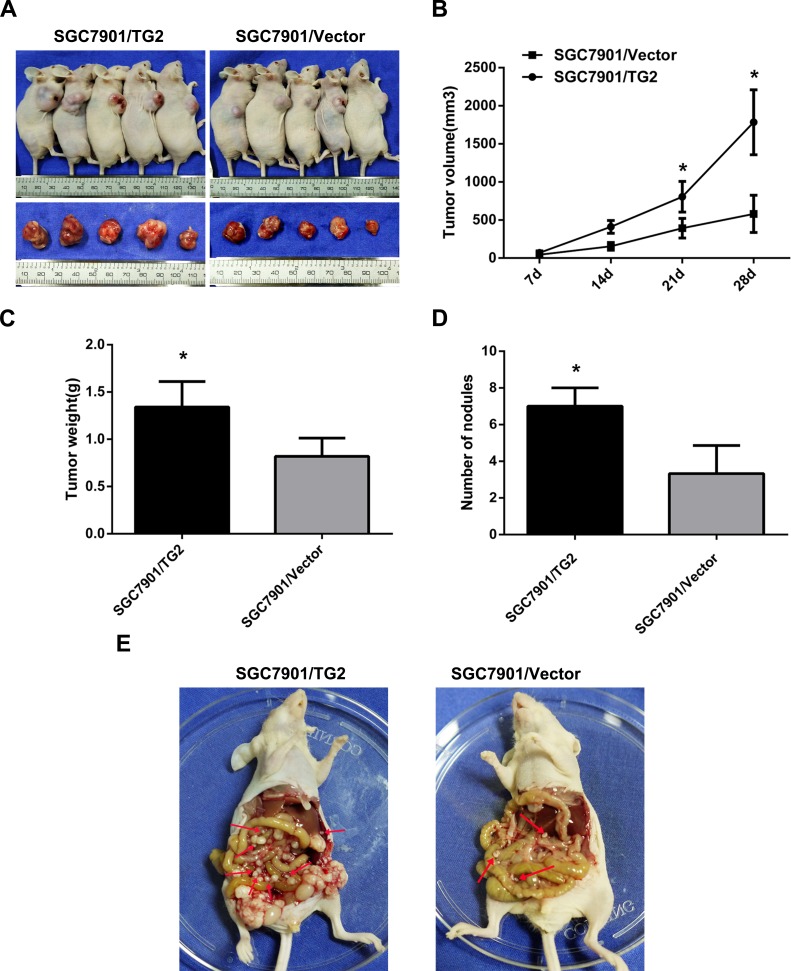
TG2 overexpression promotes subcutaneous tumor growth and peritoneal spread and metastasis in nude mice **A.** Representative photographs of nude mice tumors (*N* = 5 per group) derived from SGC7901/TG2 cells and SGC7901/Vector. **B.** Tumor volume was monitored weekly after treatment with SGC7901/TG2 cells or SGC7901/Vector (*P < 0.05). **C.** Average tumor weight derived from SGC7901/TG2 cells or SGC7901/Vector (*N* = 5) (*P < 0.05). **D.** Average peritoneal nodules from nude mice (*P < 0.05). **E.** SGC7901/TG2 cells or SGC7901/Vector were inoculated into nude mice, and peritoneal nodules (red arrows) were observed after 35 days (*N* = 5 per group). Data are means ± SD of three independent experiments, *P < 0.05.

Next, we assessed whether knockdown of TG2 repressed tumor growth and metastasis *in vivo*. Figure 5A, 5B, and 5C showed that tumor growth from MKN45/TG2-shRNA cells and NCI-N87/TG2-shRNA cells was slower than in negative controls. Moreover, average tumor weights from MKN45/TG2-shRNA and NCI-N87/TG2-shRNA cells were significantly lower than weights of tumors from MKN45/TG2-NC and NCI-N87/TG2-NC cells (MKN45/TG2-shRNA: 0.20±0.06 g; MKN45/TG2-NC: 0.46±0.12 g; MKN45/TG2-shRNA *vs*. MKN45/TG2-NC: *P* < 0.05; NCI-N87/TG2-shRNA: 0.52±0.10 g; NCI-N87/TG2-NC: 1.07±0.13 g; NCI-N87/TG2-shRNA *vs*. NCI-N87/TG2-NC: *P* < 0.05) (Figure 5D). Ki-67 antigen expression measured using IHC indicated (Figure 5E) fewer Ki-67 antigen-positive cells in tumors derived from MKN45/TG2-shRNA or NCI-N87/TG2-shRNA cells compared with tumors derived from controls (*P* < 0.05). To assess whether regulation of the ERK1/2 pathway by TG2 could be recapitulated *in vivo*, expression of TG2 and pERK1/2 were measured with IHC, and staining intensity was significantly lower in tumors derived from MKN45/TG2-shRNA or NCI-N87/TG2-shRNA cells compared with controls. In addition, treatment of nude mice with MKN45/TG2-shRNA or NCI-N87/TG2-shRNA cells inhibited formation of peritoneal metastatic nodules compared with MKN45/TG2-NC and NCI-N87/TG2-NC cells (MKN45/TG2-shRNA: 2.00±0.71; MKN45/TG2-NC: 7.40±1.14; MKN45/TG2-shRNA *vs*. MKN45/TG2-NC: *P* < 0.05; NCI-N87/TG2-shRNA: 2.40±1.14; NCI-N87/TG2-NC: 6.20±1.30; NCI-N87/TG2-shRNA *vs*. NCI-N87/TG2-NC: *P* < 0.05) (Figure 5F and 5G). Tumors grew more rapidly in the SGC7901/TG2 group and tumor weights (SGC7901/TG2: 1.34±0.27 g; SGC7901/Vector: 0.82±0.19 g; SGC7901/TG2 *vs*. SGC7901/Vector: *P* < 0.05) in this group were higher than in the SGC7901/vector group (Figure 6A, 6B, and 6C). There were significantly more visible peritoneal nodules in the SGC-7901/TG2 group compared with controls (SGC7901/TG2: 7.00±1.00; SGC7901/Vector: 3.33±1.53; SGC7901/TG2 *vs*. SGC7901/Vector: *P* < 0.05) (Figure 6D and 6E). We conclude that TG2 promotes tumorigenesis, peritoneal spread, and metastasis of GC *in vivo*.

## DISCUSSION

Recently, the significance of TG2 non-enzymatic regulation of its own activity has been established [14, 15, 24, 25], and this regulation affects many physiological processes of cell behavior such as cell growth, adhesion, migration, programmed cell death, differentiation, and ECM remodeling [14]. These cellular processes are vital to wound healing, tissue repair, and tumor growth and metastasis [26]. However, aberrant expression of TG2 and its influence on gastric cancer are unclear. Thus, we confirmed that TG2 was a critical regulator of GC progression and that expression of TG2 was frequently upregulated in GC. In addition, TG2 expression was significantly associated with the depth of tumor invasion and late TNM stages.

Multiple studies reveal that increased TG2 expression in diverse cancers and aberrant expression of TG2 are correlated with poor survival, increased drug resistance, and high tumor invasiveness. Specifically, TG2 can enhance pancreatic cancer cell motility and metastatic potential by regulating the TG2/β1 integrin/Src/uPAR pathway and EMT [27]. In breast cancer, abnormal TG2 expression can lead to glucose metabolism reprogramming, facilitating metabolic alterations of breast cancer cells, promoting drug resistance, and metastatic competence [28]. Down-regulated TG2 expression has been documented in primary tumors and upregulated expression has been confirmed in secondary metastatic tumors [29, 30]. Here, we report that TG2 promoted GC cell proliferation, migration, and invasion *in vitro* and *in vivo* with gain- and loss-of-function approaches. Furthermore, TG2 may function as an oncogene in GC and may be associated with GC progression.

TG2 can activate several pathways that contribute to its tumor-promoting properties. Increased TG2 expression is often associated with constitutive activation of NF-kB, which protects against ROS-induced cell damage, inflammatory cytokines, and chemotherapeutic drugs [7]. TG2 can also promote degradation of the phosphatase PTEN, constitutively activating the FAK/AKT pathway for regulation of cell survival and chemotherapeutic drug resistance [31]. In addition, cell surface TG2 could interact with integrins, such as β1, β3 and β5, to promote activation of the FAK/c-Raf/MEK1/2-ERK1/2 pathway [11]. Previous studies indicate that activation of the ERK1/2 pathway is needed for proliferation, migration, and invasion of tumor and non-tumor cells [32-35]. When ERK1/2 pathway activation is inhibited, the effects of umbilical cord-derived mesenchymal stem cells on breast cancer cell proliferation and migration can be reversed [36].

Here, we report that TG2 also regulated cell proliferation, migration, and invasion in GC cells. Specifically, TG2 knockdown suppressed activation of the ERK1/2 pathway, tumor growth, and peritoneal and other metastases *in vivo*, whereas TG2 overexpression reversed these changes. In addition, inhibition of the ERK1/2 pathway by U0126, a specific ERK1/2 inhibitor, could partially reverse tumor-promoting effects on proliferation, migration, and invasion caused by high TG2 expression, suggesting an involvement of the ERK1/2 pathway in regulating TG2 function in GC cells. The ERK1/2 pathway is documented to mediate tumor metastasis *via* regulating matrix metalloproteinases (MMPs) expression and activity and this plays a critical role in ECM degradation [37-39], a pivotal step during tumor metastasis [40, 41]. Thus, TG2, as an upstream regulator of ERK1/2, regulates GC cell proliferation, migration, and invasion by activating the ERK1/2 pathway. Our data provide a foundation for understanding the mechanism of TG2 overexpression in GC progression and suggest that TG2 may be a promising therapeutic target for treating GC.

## MATERIALS AND METHODS

### Ethical statement

Written consent was acquired from all participants who were fully informed of the experimental procedures during the period of research. The study was approved by the Human Research Ethics Committee of Ruijin Hospital, Shanghai Jiaotong University, School of Medicine. All experiments on animal were given permission by the Experimental Animal Ethics Committee of Ruijin Hospital and carried out according to the Guide for the Institutional Animal Care and Use Committee (IACUC) of Shanghai Jiaotong University.

### Tissue specimens

GC tissues were obtained from 127 patients, none of who received radiotherapy or chemotherapy before surgery, between 2010 and 2013 at the Ruijin Hospital, Shanghai, China. All the patients included 90 men and 37 women, and the average age was 61.1 years old. The TNM-stage of patients was determined by the UICC TNM classification. All the tissue samples were identified by clinical pathologist and then were fixed by formaldehyde and embedded by paraffin to produce tissue chips.

### Cell lines

Human GC cell lines, MKN45 and NCI-N87, were purchased from Shanghai Institutes for Biological Sciences, Chinese Academy of Sciences. Cells were cultured in the RPMI-1640 medium containing 10% calf serum with 100 U/ml penicillin and 100 U/ml streptomycin and were placed in a humidified cell incubator with 5% CO2 at 37°C.

### Quantitative real-time PCR (qRT-PCR)

Total RNA was isolated with Trizol reagent (Invitrogen, Carlsbad, CA, USA) and cDNA was synthesized with reverse transcription kit (Promega, Madison, WI, USA) following the manufacturer's instructions. QPCR was performed using an Applied Biosystems 7500 System and the SYBR Green Reagent kit (Applied Biosystems, Foster City, CA, USA) to quantitatively analyze the expression levels of TG2. The PCR primers for TG2 were 5′-CGTGACCAACTACAACTCGG-3′ (forward) and 5′-CATCCACGACTCCACCCAG-3′ (reverse). The PCR primers for GAPDH were 5′-GGACCTGACCTGCCGTCTAG-3′ (forward) and 5′-GTAGCCCAGGATGCCCTTGA-3′ (reverse).

### Western blot analysis

Cells were lysed with RIPA cell lysis buffer in the presence of protease inhibitor cocktail (Sigma, USA). The protein concentration of the cell lysates was quantified by a BCA Protein Assay Kit (Pierce, Rockford, USA). The same amount of protein samples were loaded onto 10% SDS-PAGE and then transferred onto PVDF membranes. After blocked by skim milk, the membranes were incubated in the primary antibodies diluted by TBST buffer for overnight at 4°C and then in the HRP-conjugated secondary antibody for 2-3h at room temperature. Finally the protein bands images were captured by a Tanon detection system with ECL reagent (Thermo). The primary antibodies used in the experiments were anti-TG2 (1:1000; Abcam, USA), anti-ERK1/2 (1:1000; Cell Signaling Technology, USA), anti-pERK1/2 (Thr202/Tyr204) (1:1000; Cell Signaling Technology, USA) and anti-GAPDH (1:10000; Abcam, USA).

### Immunohistochemistry (IHC)

GC tissues sections fixed by formalin and embedded by paraffin were dewaxed in xylene and rehydrated with gradient ethanol. The sections were incubated with rabbit anti-TG2 monoclonal antibody (1:150, Abcam, USA) at 4°C overnight, prior to which antigen retrieval was performed by boiling in 0.01 mol/L citrate buffer (pH 6.0). The immune complex was detected by a standard avidin-biotin detection system with the LSAB+ kit (Dako, USA). The nuclei were counterstained with hematoxylin. The sections were evaluated by three pathologists who were blinded to clinicopathologic information. TG2 staining score= positive cell score + staining intensity score. The percentage of positive cells was classified by four grades (percentage scores): 0 (0), <1/3 (1), 1/3-2/3 (2) and >2/3 (3). The intensity of staining was also divided into four grades (intensity scores): no staining (0), weak staining (1), moderate staining (2) and strong staining (3). The overall scores 0, 1-2, 3-4, and 5-6 were defined as negative (−), weak positive (±), moderate positive (+), and strong positive (++) respectively.

### Plasmids construction and transfection

The TG2 shRNA was purchased from Shanghai GeneChem Co, Ltd. The target sequence was 5′-AAATACCGTGACTGCCTTA-3′ and the negative control sequence was 5′-TTCTCCGAACGTGTCACGT-3′. The shRNA plasmid construction was hU6-MCS-Ubiquitin-EGFP-IRES-puromycin. The TG2 shRNA and negative control plasmids were transfected into MKN45 cells and NCI-N87 cells using Lipofectamine2000 (Invitrogen, Carlsbad, CA, USA), respectively. Then the stable transfected cell lines (MKN45/TG2-shRNA, MKN45/TG2-NC, NCI-N87/TG2-shRNA, NCI-N87/TG2-NC) were screened by using puromycin and western blot was performed for identification.

The full length TG2 cDNA was obtained by RT-PCR from total RNA of GC samples. The primer sequences of TG2 were 5′-ACGGGCCCTCTAGACTCGAGCGCCACCATGG CCGAGGAGCTGGTCTTAGAG-3′ (forward), 5′-AGTCCAGTGTGGTGGAATTCGGCGGGG CCAATGATGACATTCC-3′ (reverse) and subcloned into CMV-MCS-3FLAG-SV40-Neomycin plasmid (GeneChem, Shanghai, China). The constructed plasmid and empty vector were transfected into SGC-7901 and AGS cells using Lipofectamine 2000 (Invitrogen, Carlsbad, CA, USA) in accordance with the manufacturer's protocol. Stable clones (SGC7901/TG2, SGC7901/Vector, AGS/TG2, AGS/Vector) were selected by using G418 (1.2 mg/ml; Gibco, New York, USA).

### Cell proliferation assay

Cells (2×10^3^/well) were seeded into 96-well plates for 4 days, and cell proliferation was measured by spectrophotometer at 0h, 24h, 48h, 72h and 96h using a Cell Counting Kit-8 (Dojindo, Kumamoto, Japan) according to the manufacturer's instructions.

### Cell migration and invasion assay

Cell migration and invasion assays were assessed using transwell chambers (Corning Costar, NY, USA). Cells were cultured in serum-free RPMI-1640 for 12-16h, and then 1×10^5^ cells in 200ul serum-free medium were seeded into the upper chamber, and 800ul medium with 10% fetal calf serum was added to the lower chamber. For the invasion assay, the insert membranes were coated with diluted matrigel (BD Bioscience, CA, USA). After cultured for 24h, cells that did not move through the membranes were removed and then the membranes were stained with 0.1% crystal violet for 30 min. The stained cells were counted and photographed using an inverted microscope.

### *In vivo* tumorigenesis and metastasis

Four-week-old male BALB/C nude mice, which were purchased from the Institute of Zoology, Chinese Academy of Sciences and housed at a specific pathogen-free environment, were injected subcutaneously with 6×10^5^ gastric cancer cells in 100μl PBS or inoculated peritoneally with 2×10^6^ cells in 250μl PBS. Tumor length (L) and width (W) were measured every week. Tumor volume was evaluated using the following formula: tumor volume =LW^2^π/6. All mice were sacrificed after 30 days. Subcutaneous tumor grafts were excised and weighed, and peritoneal metastasis nodules were counted and analyzed.

### Statistical analysis

The experimental results were analyzed by SPSS 18.0 software and shown as mean ± standard deviation (SD). The relationship between the expression level of TG2 and clinicopathologic parameters was examined by the Pearson χ2 test or Fisher's exact tests. The differences between the two groups were calculated by the Student's t test. The significance level was set at *P* < 0.05.
